# Functional Magnetic Graphene Composites for Biosensing

**DOI:** 10.3390/ijms21020390

**Published:** 2020-01-08

**Authors:** Fan Li, Yan Huang, Kai Huang, Jing Lin, Peng Huang

**Affiliations:** Marshall Laboratory of Biomedical Engineering, International Cancer Center, Laboratory of Evolutionary Theranostics (LET), School of Biomedical Engineering, Shenzhen University Health Science Center, Shenzhen 518060, China; lifanrj@163.com (F.L.); huangyan_shenzhen@163.com (Y.H.); huangkai@u.nus.edu (K.H.); jingl@szu.edu.cn (J.L.)

**Keywords:** magnetic nanoparticles, graphene, chemical sensors, biological sensors, electronic sensors

## Abstract

Magnetic graphene composites (MGCs), which are composed of magnetic nanoparticles with graphene or its derivatives, played an important role in sensors development. Due to the enhanced electronic properties and the synergistic effect of magnetic nanomaterials and graphene, MGCs could be used to realize more efficient sensors such as chemical, biological, and electronic sensors, compared to their single component alone. In this review, we first reviewed the various routes for MGCs preparation. Then, sensors based on MGCs were discussed in different groups, including optical sensors, electrochemical sensors, and others. At the end of the paper, the challenges and opportunities for MGCs in sensors implementation are also discussed.

## 1. Introduction

As a star material, graphene has attracted much attention since its discovery in 2004 [1]. This exciting two-dimensional (2D) material with single-atom-thick, exhibited several exceptional and unique physical and chemical properties, such as exceptional electrical conductivity, high specific surface area, and biocompatibility. Thus, graphene was used to implement applications ranging from physical devices construction to sensor development and cancer theranostics in recent years [2]. Additionally, magnetic nanoparticles, due to their magnetic, electrical, catalytic and optical properties were widely employed as building blocks in sensors [3]. For example, the electrocatalysis activity of magnetic nanoparticles surface was used to develop signal-amplification sensors of small molecules (e.g., H_2_O_2_, Nicotinamide adenine dinucleotide (NADH) or O_2_) [4]. Therefore, the marriage of graphene and magnetic nanoparticles could generate one new kind of hybrid material, magnetic graphene composites (MGCs), which show great potential in the construction of sensors [5].

Compared with either nanomaterial alone, MGCs exhibited additional unique physicochemical properties, such as better electronic conductivity, better stability for biomolecules and large surface area for molecules immobilization and so on [2]. In the past few years, MGCs were widely used for developing advanced sensors, which were implemented to detect various types of analytes, including ions [6], small molecules [7,8], nuclear acids [9], other biomacromolecules [10] and cells [11]. In this review, we will systematically summarize the fabrications of magnetic graphene composites (MGCs) sensors. Then the different kinds of sensors based on composites categorized with output signals were reviewed. After that we will discuss and conclude the challenges and opportunities of sensors with MGCs.

## 2. Fabrication of Magnetic Graphene Composites (MGCs) for Sensors

The construction of magnetic graphene hybrid nanostructures for sensors is generally realized in four different methods: ex situ non-covalent assembly, ex situ covalent assembly, in situ reductions and in situ hydrothermal synthesis, as shown in Table 1. In addition, there were other preparation strategies including in situ sol–gel synthesis, in situ microwave-assisted synthesis and more, which were also used to prepare MGCs for other applications. However, in this section, we focus on the first four kinds of construction methods.

### 2.1. Ex Situ Assembly Methods

For ex situ assembly, graphene or its derivatives and magnetic nanoparticles were synthesized separately in advance and then been conjugated together by non-covalent or covalent interactions.

#### 2.1.1. Non-Covalent Assembly Methods

Nov-covalent conjugation was a standard assembly method for MGCs building. For instance, Zhang et al. implemented non-covalent conjugation between graphene oxides (GOs) with magnetic microbeads based on specific antibody binding reaction [12]. In this system, anti-EpCAM antibodies modified GOs and anti-lgG antibodies modified magnetic microbeads were prepared based on the electrostatic interaction between proteins on the surfaces of GOs and microbeads. Then immune-magnetic GO composites were synthesized through the interaction of anti-EpCAM antibodies and anti-lgG antibodies. In addition, the chitosan could also be used as the bridge for a non-covalent combination between graphene oxides (GOs) with Fe_3_O_4_ nanoparticles [13,14,15]. Others noncovalent bonds, including van der Waals interaction, π–π staking, electrostatic interactions and hydro bonding were also used to building MGCs [10,16,17,18,19,20]. Normally, the energies of individual noncovalent interactions were lower than the covalent bonds. However, the combined noncovalent interactions based on numerous binding sites showed rivaled energy compared with some covalent bonds [21].

#### 2.1.2. Covalent Assembly Methods

Alternatively, magnetic nanoparticles could bind with graphene or its derivatives via covalent coupling reaction including amidation reaction and click chemistry [22,23,24,25,26,27,28,29,30,31]. For example, Bi et al. reported magnetic GO-hemin composites probes based on amidation reaction [22]. The thiol-modified magnetic nanoparticles were coated with NH_2_ groups by reacting with S-2-pyridylthio cysteamine hydrochloride. Meanwhile, the carboxylic groups on the surface of GO were activated by 1-ethyl-3-(3-(dimethylamino) propyl)carbodiimide (EDC) and *N*-hydroxysuccinimide (NHS). Then the magnetic GO composites were formed based on an amidation reaction between the activated carboxylic groups with the NH_2_ groups. As mentioned above, click chemistry was also used to assemble MGCs. For instance, alkynyl-functionalized GO was prepared by propargylamine hydrochloride reagent. Meanwhile, azide-functionalized magnetic silica nanoparticles and alkynyl- functionalized Ab2 were obtained through reactions. Just by mixing them together, the Ab2/MSN/GO composites were formed through click chemistry [31].

In this way, for the fabrication of MGCs, the size and shape of involved nanoparticles could be controlled accurately. However, due to limited density and non-uniform distribution of functional groups on GO or magnetic nanoparticles surfaces, it was difficult to synthesized high loading and uniform MGCs.

### 2.2. In Situ Assembly Methods

#### 2.2.1. In Situ Reduction Methods

Most MGCs were built via in situ reduction of metal precursors on the surface of GO [32,33,34,35,36,37,38,39,40,41,42,43,44,45,46,47,48,49,50,51,52,53,54,55,56,57,58,60,61,62,63,64,65,66,67,68,69,70,71,72,73,74,75,76,77,79,80]. For magnetic materials, metal precursors, including FeCl_2_, FeCl_3_, Fe(acac)_3_, (NH_4_)_2_Fe(SO_4_)_2_ and more, which would bind with GO surfaces for electrostatic interactions between positive charges of metal ions with negative charges of groups on GO surfaces. With the help of reductants, such as NaOH, amines, hydrazine hydrate and NaBH_4_ agents, magnetic nanoparticles were synthesized on GO surfaces in situ. For example, Fe_3_O_4_-Pd/RGO nanocomposites were prepared by in situ reduction of FeCl_2_ and PdCl_2_ with NaOH under pH 13.0 condition [32], and Fe_3_O_4_-Pd/RGO composites were synthesized by the reduction of (NH_4_)_2_Fe(SO_4_)_2_ and NH_4_Fe(SO_4_)_2_ with NH_4_OH under pH 11.5 condition [36].

The in situ reduction method was almost the same as the conventional synthesis processes of metal nanoparticles in solution alone with high efficiency. However, the sizes of magnetic nanoparticles were difficult to control.

#### 2.2.2. In Situ Hydrothermal Synthesis Methods

Hydrothermal synthesis method was not a popular one for construction of MGCs for sensing [7,78]. For instance, Zhao et al. reported Fe_3_O_4_/GO-CNT composites based on hydrothermal treatment [7]. After treated with HNO_3_, the carbon nanotubes (CNT) were adsorbed onto the GO surfaces to form GO/CNT composites. After hydrothermal treatment, ferric ions were reduced to form Fe_3_O_4_ nanoparticles on the GO/CNT surfaces in situ [7].

## 3. Sensors Based on MGCs

Compared with graphene or magnetic nanoparticles alone, MGCs exhibited additional physicochemical properties. For example, these nanocomposites showed high specific surface area, excellent optical properties, remarkable catalytic properties, and outstanding electrical properties. In this section, the current state of sensors based on MGCs in optical, electrochemical, and other transduction methods will be reviewed in detail, as shown in Table 1.

### 3.1. Optical Sensors

**Figure 1:**
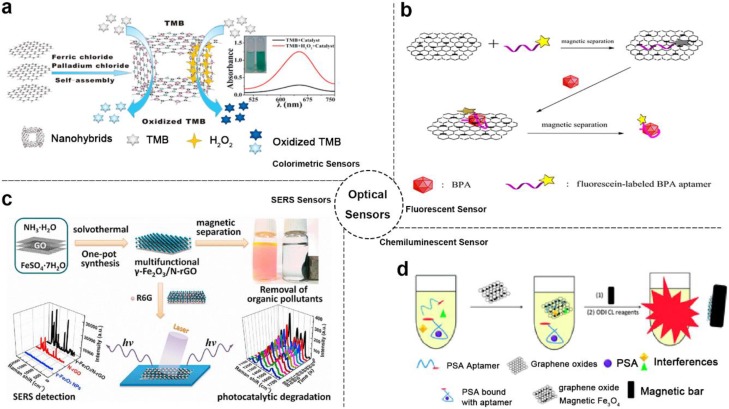
Optical sensor-based MGCs: (**a**) Schematic diagram of colorimetric sensor example. Adapted with permission from ref. [32]. Copyright © 2015, American Chemical Society; (**b**) Schematic diagram of fluorescent sensor example. Adapted with permission from ref. [35]. Copyright © 2014, American Chemical Society; (**c**) Schematic diagram of SERS sensor example. Adapted with permission from ref. [47]. Copyright © 2013, Elsevier; (**d**) Schematic diagram of chemiluminescent sensor example. Adapted with permission from ref. [37]. Copyright © 2015, Royal Society of Chemistry.

With using the enhanced catalytic and optical properties of MGCs, several kinds of optical sensors were developed including colorimetric, fluorescence, surface-enhanced Raman scattering (SERS) and more [22,32,33,78,81]. For instance, Zheng et al. reported a colorimetric probe based on 3D graphene-magnetic palladium nanohybrids (Fe_3_O_4_-Pd/3DRGO), as shown in Figure 1a [32]. In this system, Fe_3_O_4_-Pd/3DRGO demonstrated enhanced peroxidase catalytic activity and high affinity toward substrate H_2_O_2_ with a synergistic effect between graphene with magnetic nanoparticles. According to these features, a highly sensitive and selective colorimetric sensor for glutathione (GSH) (detection limits of detection (LOD): 5.2 × 10^−8^ M) and glucose (LOD: 1.3 × 10^−7^ M) was realized. Moreover, this colorimetric method could be used to detect glucose in urine with LOD of 6.8 mM (72.9 mg dL^−1^). In a similar way, MGCs were used to implement colorimetric analysis of target cancer cells [34].

As known, GO and magnetic nanoparticles were both strong fluorescence quenching agents. Based on these materials, numerous fluorescent sensors were developed. Combining the GO and magnetic nanoparticles, more excellent fluorescent sensors based on MGCs could be complemented [35,79]. As shown in Figure 1b, Hu et al. fabricated an outstanding magnetic separate “turn-on” fluorescent sensor based on magnetic graphene nanocomposites and aptamer for bisphenol A (BPA) [35]. Beside the fluorescence quencher role, the composites served as a separation medium for enhancing the fluorescence signal. Based on this sensor, LOD of 0.071 ng mL^−1^ was obtained, which is lower than some other BPA sensors [82]. In addition, the composites probes could be recycled easily based on magnetic properties.

Moreover, the MGCs could also be used to promote the development of SERS sensors. In previous studies, graphene already exhibited potential in SERS analysis for its novel features. Combining the properties of magnetic nanoparticles, MGCs showed more excellent SERS detection ability compared with graphene alone (Figure 1c) [47]. Based on the MGCs of γ-Fe_2_O_3_/rGO, the detection limit of the SERS sensor toward R6G was low as to 5 × 10^−7^ M. In addition, this composite also exhibited removal and degradation ability of organic pollutants based on its separation, adsorption, and photocatalytic properties.

Additionally, MGCs realized chemiluminescent or electro-chemiluminescent analysis methods using the properties of the high specific surface area, magnetic separation and more together [8,15,36,37,38,49,83,84]. As shown in Figure 1d [37], a chemiluminescent aptasensor based on Fe_3_O_4_/GO composites and prostate specific antigen (PSA) aptamers was developed for PSA detection with LOD of 0.5 ng·mL^−1^.

### 3.2. Electrochemical Sensors

Compared with other sensors, electrochemical sensors displayed the advantages of high sensitivity, good stability, and reproducibility, which attracted the most attention. As shown in Table 1, most MGCs-based sensors were electrochemical sensors.

Because of its excellent electrical conductivity and other features, graphene was already proved to be an ideal material for electrochemical sensors, compared with other carbon-based materials [85]. Combining the unique properties of magnetic materials with graphene and synergistic effects of two components, such as better electronic conductivity and better stability for biomolecules as well as large surface area for molecules immobilization, MGCs gained increasing attention in the development of electrochemical sensors, including sensing of metal ions, H_2_O_2_, glucose, amino acids, proteins, DNA, viruses, and more.

Heavy metal ions were recognized as an environmentally hazardous agents. Determination methods development of trace amounts of them in different samples using MGCs was important for our public health. For instance, Chimezie et al. reported an electrochemical sensor for As(III) determination with magnetic reduced graphene oxides composites modified screen-printed electrode (rGO-Fe_3_O_4_/SPE) [17]. This MGCs functional electrochemical sensor exhibited a low detection limit of 0.1 μg L^−1^ for As(III). Moreover, the sensor showed its excellent stability for real water samples tests, including lake water, mineral water and reversed osmosis drinking water. In addition, other electrochemical sensors with MGCs were used to trace analysis of europium ions, chromium ions and more [23,53].

**Figure 2:**
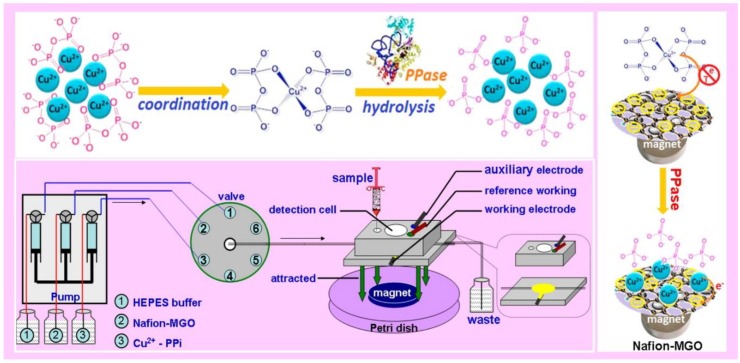
Schematic diagram of microfluidic chemical device for pyrophosphatase activity monitoring. Adapted with permission from ref. [55]. Copyright © 2015, American Chemical Society.

Moreover, numerous toxins and pesticides could also cause severe environmental and safety issues. By using acetylcholinesterase (AChE) magnetic graphene nanocomposites (Fe_3_O_4_/GO/AChE MNCs), Liang et al. fabricated a replaceable on-chip enzymatic microreactor for electrochemical detection of dimethoate [55]. As shown in Figure 2, Fe_3_O_4_/GO/AChE MNCs could be flushed out easily and quickly based on magnetic separation properties of the composites. In addition, the high specific surface area property supported composites to load more AChE molecules for improving the sensitivity of dimethoate analysis with LOD of 0.18 μg·L^−1^.

Meanwhile, for revealing some biological or clinical processes, the determination and monitoring of some small molecules are urgently needed, such as H_2_O_2_, glucose, amino acids, hormones, and more [18,24,28,60,63,80]. For this aspect, Xin et al. implemented determination of H_2_O_2_ by using graphene sheets (GS)-Nafion film and Fe_3_O_4_-Au nanoparticles coated horseradish peroxidase (HRP) modified SPE (SPE/GS-Nafion/Fe_3_O_4_-Au-HRP) electrode, which showed a low LOD of 1.2 × 10^−5^ mol·L^−1^ [16]. On the basis of biocompatibility of MGCs, the loaded enzymes played important roles for the enhanced biosensor, which implemented ultrasensitive analysis of different target molecules [28,80,86]. In addition, Naghib et al. realized ultrasensitive non-enzymatic sensing of glucose using rGO- Fe_3_O_4_-gelatin modified glassy carbon electrode [54].

Additionally, as mentioned previously, micro-biomolecules biosensor realization based on MGCs, such as proteins, DNA and more, received significant attention due to the important roles of these molecules in the clinic [74]. For example, Lin et al. reported magnetic graphene oxide modified Au electrode for cancer diagnosis based on vascular endothelial growth factor (VEGF) determination [51]. This sensor provided higher sensitivity and a broader range of linear detection, even compared to ELISA kit. Moreover, Jahanbani et al. designed a label-free DNA electrochemical sensor based on MGCs (Fe_3_O_4_NP-RGO) and 1-pyrenebutyric acid-*N*-hydroxysuccinimide ester (PANHS) modified electrode [61]. As shown in Figure 2, Lin et al. reported a novel chemical assay platform for monitoring pyrophosphatase (PPase) activity based on magnetic graphene nanosheet composites [50].

**Figure 3:**
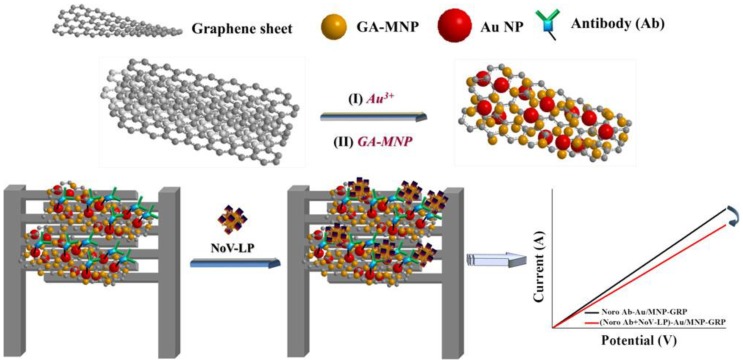
Schematic diagram of electrochemical sensors for a norovirus-like particle. Adapted with permission from ref. [20]. Copyright © 2017, American Chemical Society.

Lastly, MGCs also played an important role in electrochemical sensors of pathogenic microorganisms, such as bacteria and viruses [20]. As shown in Figure 3, the gold/magnetic nanoparticles decorated graphene (Au/MNP-GRPs) composite was used to implement a chemical sensor for the norovirus-like particles (NoV-LP) [20]. Due to the magnetic properties, the Pt-interdigitated electrode was easily modified with the Au/MNP-GRPs composite, which was used to load antibody for detection of NoV-LP.

### 3.3. Other Sensors

Except for the sensors mentioned above, some novel sensors were implemented based on MGCs. For instance, Chen et al. reported a polycrystalline silicon nanowire field-effect transistor (poly SiNW-FET) which was modified by magnetic graphene with long chain acid groups (MGLA) [75]. The MGLA/poly-SiNW-FET biochip showed potential for cancer biomarker diagnosis in the clinic, which worked well in human urine samples.

**Figure 4:**
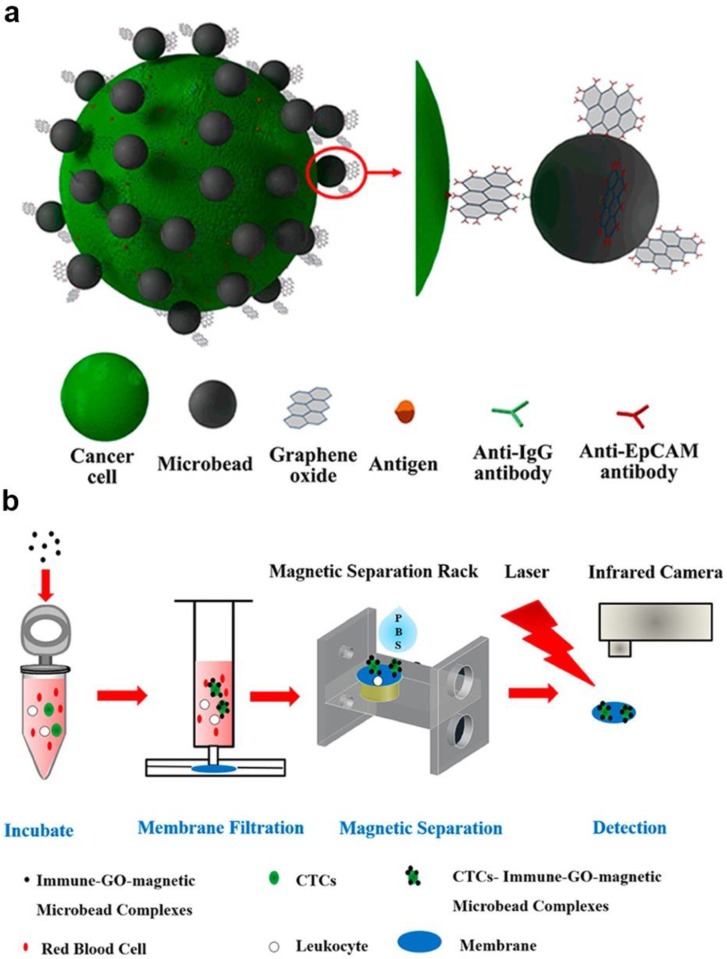
Novel sensors with photothermal way-based MGCs: (**a**) Schematic diagram of fabrication of the composite (**b**) Schematic diagram of photothermal sensor work process. Adapted with permission from ref. [12]. Copyright © 2016, American Chemical Society.

Moreover, the photothermal effect was also used in fabricating sensors. For example, Zhang et al. developed a sensitive, portable, and cost-effective detection method with immune-GO-magnetic nanoparticle nanohybrids; these nanohybrids were used to implement a thermal sensor (Figure 4) [12]. Under laser irradiating, the GOs converted the energy into heat and caused temperature increasing, thus could be used to detect targeted cells by monitoring the temperature. In this biosensor, the environment condition, properties of containers and some factors could influence the biosensor’s performance. However, they provided new idea for the signal transduction. Moreover, a novel surface plasmon resonance (SPR) was realized with PDA-Ag@Fe_3_O_4_/rGO [87].

In addition, a zetasizer system based on GO/Fe_3_O_4_@GSH nanocomposites was reported for the determination of As(III)As(V) species in real samples [30]. At the same time, the GO/Fe_3_O_4_@GSH showed higher adsorption capacity of As(III) compared with other materials. Similar to the strategy of using magnetic separation properties, MGCs were used to promote the traditional analysis methods, such as liquid chromatography-mass spectrometry (LC-MS), ultraviolet (UV), gas chromatography-mass spectrometry (GC-MS), and more [7,27,76].

## 4. Summary and Outlook

In this review, the fabrications of MGCs and numerous kinds of related sensors were discussed in detail. Through non-covalent and covalent binding between graphene with magnetic nanoparticles to in situ synthesis of magnetic nanoparticles on graphene surfaces, a significant number of MGCs were successfully fabricated. Based on these composites and excellent properties of them, researchers realized many types of sensors in different ways, including colorimetric, fluorescent, photothermal, electrochemical, and more. These sensors showed enhanced selectivity and sensitivity as well as a combination of properties.

The synergistic advantages of MGCs broadened the application of sensors in many fields. However, the MGCs remain in its early stage, numerous challenges need to be solved. Up to now, MGCs preparation with controllable sizes, shapes and low-cost, high-yield manner remain the bottlenecks. Moreover, more precise controlling of specific arrangement of magnetic materials and graphene would improve the performance of MGCs in sensing. In conclusion, the MGCs will bring more synergistic advantages to sensor-building and improve the performance of current methods.

## Figures and Tables

**Table 1:** Sensors based on the use of magnetic graphene composites.

Preparation Method	Composite	Sensor Type	Analyte(s)	Linear Dynamic Range (LDR)	Limit of Detection (LOD)	Real Sample (s)	Citation
Ex situ non-covalent conjugation Assembly	CS/Fe_3_O_4_/GO/T-Apt/HM	Chemiluminescence	Thrombin	5.0 × 10^−15^–2.5 × 10^−10^ M	1.5 × 10^−15^ M	Serum	[15]
	BGNs- Fe_3_O_4_/Au-Ab_1_	Electro-chemiluminescent immunoassay	Tetrodotoxin	0.01–100 ng·mL^−1^	0.01 ng·mL^−1^	Muscle samples	[10]
	SPE/GS-Nafion/Fe_3_O_4_-Au-HRP	Electrochemical	H_2_O_2_	2.0 × 10^−5^–2.5 × 10^−3^ M	1.2 × 10^−5^ M	Contact lens care solution	[16]
	rGO-Fe_3_O_4_/SPE	Electrochemical	As(III)	2–300 μg·L^−1^	0.10 μg L^−1^	Lake, reverse osmosis and natural mineral water samples	[17]
	HRP-GS-Fe_3_O_4_-CS/GC	Electrochemical	H_2_O_2_	2.49 × 10^−5^–1.67 × 10^−3^ M	3.05 × 10^−6^ M	-	[18]
	Fe_3_O_4_@PDA-rGO	Electrochemical immunoassay	Microcystin-LR	0.01–50 mg·L^−1^	0.007 ug·L^−1^	Real water	[19]
	Fe_3_O_4_-GO	Photothermal Imaging	Cancer cell	100–700 cells	100 cells	Human Blood	[12]
	Fe_3_O_4_@Chitosan/GO	Flame atomic absorption spectrometer (FAAS)	Lead ion Pb^2+^	10–800 ng·mL^−1^	2 ng·mL^−1^	Blood	[13]
Ex situ covalent conjugation Assembly	Fe_3_O_4_-GO-hemin	Colorimetry	Glutathione (GSH)	10^−10^–10^−6^ M	8.2 × 10^−11^ M	Extraction of Ramos cells	[22]
	Pd/Fe_3_O_4_-PEI-rGO	Colorimetry	H_2_O_2_	0.5–150 × 10^−6^ M	0.1 × 10^−6^ M	-	[26]
	rGO-Fe_3_O_4_@Silance-rGO	Electrochemical	Europium metal ion	Differentialpulsestrippingvoltammetry (DPSV) (0.99–29.12 μg·L^−1^); Squarewavestrippingvoltammetr (SWSV) (0.059–47.6 μg·L^−1^)	DPSV (0.30 μg·L^−1^); SWSV (0.019 μg·L^−1^)	Water, food, urine and human blood serum	[23]
	Ag-Fe_3_O_4_-GO/GCE	Electrochemical	Nitrite	0.5 × 10^−6^ M–0.72 × 10^−3^ M, 0.72–8.15 × 10^−3^ M	0.17 × 10^−6^ M	Tap water	[25]
	Fe_3_O_4_-rGO-GOx	Electrochemical	Glucose	0.05–1 × 10^−3^ M	0.1 × 10^−6^ M	-	[28]
	Fe_3_O_4_-rGO	Electrochemical	Lobetyolin	1.0 × 10^−7^–1.0 × 10^−4^ mol·L^−1^	4.3 × 10^−8^ M	Radix Codonopsis	[29]
	Bio-Ab-Cor/AuNPs/ Fe_3_O_4_-rGO@Nafion/GCE	Electrochemical immunoassay	Cortisol	0.1–1000 ng·mL^−1^	0.05 ng·mL^−1^	Human serum	[24]
	Fe_3_O_4_@SiO_2_-GO	Electrochemical immunoassay	Cancer antigen 153	10^−3^–200 U·mL^−1^	2.8 × 10^−4^ U·mL^−1^	Serum	[31]
	Fe_3_O_4_/GO@GSH	Zeta potential analyzer	As(III)	0.5–1.5 mol·L^−1^	0.1 mg·L^−1^	Tea samples	[30]
	TETA-Fe_3_O_4_-GO	MSPE-LC-MS/MS	Estrogens	-	0.15–1.5 ng·L^−1^	Tap water, well river, river water	[27]
In situ Reduction	Fe_3_O_4_-Pd/3DRGO	Colorimetry	GSH/Glucose	0.4–40 × 10^−6^ M/0.5–60 × 10^−6^ M	5.2 × 10^−8^ M/1.3 × 10^−7^ M	Human urine	[32]
	Fe_3_O_4_/NG	Colorimetry	H_2_O_2_ and Glucose	17.1 × 10^−6^ M–10 × 10^−3^ M 17.1 × 10^−6^ M~18.0 × 10^−3^ M	17.1 × 10^−6^ M 57.9 × 10^−6^ M	-	[33]
	Fe_3_O_4_/GO/PtNPs	Colorimetric	Breast cancer cells	100–1000 cells	100 cells	-	[34]
	Aptamer- Fe_3_O_4_/GO	Fluorescent	Bisphenol A	0.2–10 ng·mL^−1^	0.071 ng·mL^−1^	Actual water	[35]
	Fe_3_O_4_-Chitosan-GO	Fluorescence spectroscopy, and MALDI-MS	Bacterial cell	P.aeruginosa, 4–40 × 10^2^ cfu·mL^−1^ S.aureus, 1–30 × 10^2^ cfu·mL^−1^	1.0–4.0 × 10^2^ cfu·mL^−1^	Blood colloids	[14]
	ILs-Fe_3_O_4_@DA/GO/β-CD	Chemiluminescence	Lysozyme	1.0–80 × 10^−9^ mg·mL^−1^	3.0 × 10^−10^ mg·mL^−1^	Human urine	[36]
	Fe_3_O_4_/GO	Chemiluminescence	prostate specific antigen (PSA)	1.6–50 ng·mL^−1^	0.5 ng·mL^−1^	25% human serum.	[37]
	β-CD/Cs- Fe_3_O_4_/GO-SMIP	Chemiluminescence	Bovine serum albumin (BSA)	5.0 × 10^−7^–1.0 × 10^−4^ mg·mL^−1^	1.1 × 10^−7^ mg·L^−1^	-	[38]
	Fe_3_O_4_/rGO-MWCNTs/SMIP	Chemiluminescence	Lysozyme	5.04 × 10^−9^–4.27 × 10^−7^ g·mL^−1^	1.90 × 10^−9^ g·mL^−1^	Eggs	[39]
	Fe_3_O_4_/GO/IL/PBA	Chemiluminescence	Horseradish peroxidase	1.0 × 10^−4^–8.0 × 10^−3^ mg·mL^−1^	2.9 × 10^−5^ mg·mL^−1^	Waste water	[40]
	Si/Fe_3_O_4_/GO/MIP	Chemiluminescence	Dopamine	8.0–200.0 ng·mL^−1^	1.5 ng·mL^−1^	Urine	[41]
	Fe_3_O_4_/GO/Ag/AgCl	Chemiluminescence	Nitrite	5–200 ng·mL^−1^	1.15 ng·mL^−1^	Sausage	[42]
	Fe_3_O_4_@POM/rGO/ Ru(bpy)_3_^2+^	Electro-chemiluminescent	Nicotinamide adenine dinucleotide (NADH), L-lactate dehydrogenase	5 × 10^−9^ M–5 × 10^−4^ M for L-lactate	0.1 × 10^−9^ M for NADH; 0.4 × 10^−9^ M for L-lactate	Serum	[43]
	Fe_3_O_4_/GO	Electro-chemiluminescent	Thrombin	2.0–50 × 10^−9^ mol·L^−1^.	1.3 × 10^−9^ mol·L^−1^	-	[44]
	Fe_3_O_4_/GO/Ab_2_/Ru(bpy)_3_^2+^	Electro-chemiluminescent immunoassay	3,30,5-triiodothyronine (T3)	0.1 pg·mL^−1^–10 ng·mL^−1^	0.03 pg·mL^−1^	-	[45]
	Fe_3_O_4_/GNs	Electro-chemiluminescent immunoassay	PSA	0.003–50 ng·mL^−1^	0.72 ng·mL^−1^	Human serum	[46]
	γ-Fe_2_O_3_/rGO	SERS ^1^	R6G molecules	5 × 10^−7^–5 × 10^−4^ M	5 × 10^−7^ M		[47]
	Fe_3_O_4_/GO/Au	SERS ^1^	Thiocyanate (SCN^−^)	-	10^−8^ g·L^−1^	Milk	[48]
	Fe_3_O_4_/RGO	Electrochemical	Folate receptor	0.01–100 ng·mL^−1^	7.8 pg·mL^−1^	Pathological serum samples	[49]
	Fe_3_O_4_/GO	Electrochemical	Pyrophosphatase	0.1–20 mU·mL^−1^	0.05 mU·mL^−1^	-	[50]
	Fe_3_O_4_/GO	Electrochemical	Vascular endothelial growth factor (VEGF)	31.25–2000 pg·mL^−1^	31.25 pg·mL^−1^	Plasma	[51]
In situ Reduction	Fe_3_O_4_/GO/GC	Electrochemical	H_2_O_2_, NADH, Lactate, Ascorbicacid(AA), Dopamine(DA) Uric acid(UA) Nitrite	H_2_O_2_, 2 × 10^−8^–2.8 × 10^−7^ M; NADH, 2 × 10^−6^–1.5 × 10^−5^ M; Lactate, 2 × 10^−4^–2.2 × 10^−3^ M; AA, 1.6 × 10^−4^–7.2 × 10^−3^ M; DA, 4 × 10^−7^–3.5 × 10^−6^ M; UA, 4 × 10^−6^–2 × 10^−5^ M; Nitrite, 1 × 10^−6^–9.2 × 10^−5^ M	H_2_O_2_, 6 × 10^−9^ M; NADH, 4 × 10^−7^ M; Lactate, 2 × 10^−4^–2.2 × 10^−3^ M; AA, 2 × 10^−5^ M; DA, 8 × 10^−8^ M; UA, 5 × 10^−7^ M; Nitrite, 3 × 10^−7^ M;	Real samples for Nitrite	[52]
	Fe_3_O_4_/GO	Electrochemical	Chromium (Cr^+3^)	0.2–2 × 10^−9^ M	-	-	[53]
	Fe_3_O_4_/GO/Gelatin	Electrochemical	glucose	0.1–10 × 10^−3^ M	0.024 × 10^−6^ M	Human blood	[54]
	Fe_3_O_4_/GO/AChE	Electrochemical	Organophosphorus pesticide	1–20 μg·L^−1^	0.18 μg·L^−1^	-	[55]
	FePc@ Fe_3_O_4_/rGO	Electrochemical	Tert-butyl hydroperoxide (TBHP)	20 × 10^−6^ M–60 × 10^−3^ M	7.5 × 10^−6^ M	Cosmetic sample	[56]
	Fe_3_O_4_/GO/β-CD/GCE	Electrochemical	Tryptophan	5.0 × 10^−7^ M–7.5 × 10^−4^ M	3.1 × 10^−7^ M	-	[57]
	Ni-PDA/CNTs/GO/ Fe_3_O_4_/CPE	Electrochemical	Salicylic acid (SA)	5.00–155 × 10^−6^ M	900 × 10^−9^ M	Water	[58]
	Fe_3_O_4_/GO/CNT	Electrochemical	Diclofenac (DCF)	100–1300 × 10^−12^ M	33 × 10^−12^ M	Diclofenac sodium ampoule	[59]
	Fe_3_O_4_/GQDs/GCE	Electrochemical	Amino acid	L-Cys (0.01–100 × 10^−6^ M); L-Tyr (0.09–230 × 10^−6^ M); L-Asp (1–50 × 10^−6^ M): L-Phe (0.5–650 × 10^−6^ M)	L-Cys (0.01 × 10^−6^ M); L-Tyr (0.09 × 10^−6^ M); L-Asp (1 × 10^−6^ M): L-Phe (0.5 × 10^−6^ M)	-	[60]
	Fe_3_O_4_/rGO-GCE	Electrochemical	DNA sequences	1.0 × 10^−18^–1.0 × 10^−8^ M	2.8 × 10^−19^ M	Genomic samples extracted from blood	[61]
	Fe_3_O_4_/rGO-GCE	Electrochemical	Phenylalanine	100–1000 × 10^−9^ M	14.5 × 10^−9^ M	-	[62]
	Fe_3_O_4_/rGO/GOx-GCE	Electrochemical	Glucose	0.05–1.5 × 10^−3^ M	0.15 × 10^−6^ M	Human serum	[63]
	Fe_3_O_4_@ZIF-8/rGO/GCE	Electrochemical	Dopamine	2.0 × 10^−9^–1.0 ×10^−5^ M	6.67 × 10^−10^ M	Urine and serum	[64]
	β-CD- Fe_3_O_4_/rGO	Electrochemical	Tetracycline and doxycycline	0.5–90.0 ng·L^−1^	0.18 ng·L^−1^	Milk	[65]
In situ Reduction	Fe_3_O_4_/GQDs/MWCNTs/GCE	Electrochemical	Progesterone	0.01–0.5 and 0.5–3.0 × 10^−6^ M	2.18 × 10^−9^ M and 16.84 µA M^−1^	Human serum	[66]
	UA/FePtGNR/SPCE	Electrochemical	Ampyra (4-aminopyridine or dalfampridine)	0.08–9.0 × 10^−6^ M	0.028 × 10^−6^ M	Biological fluids	[67]
	β-CD/Au/Fe_3_O_4_/GO/GCE	Electrochemical	Sunset yellow	5.0 × 10^−9^–2 × 10^−6^ M	2 × 10^−9^ M	Water sample and mirinda drink	[68]
	Fe_3_O_4_/GO/Ag/AuNPs/MIPs	Electrochemical	Dibutyl phthalate (DBP)	2.5 × 10^−9^–5 × 10^−5^ M	8 × 10^−10^ M	Drink samples	[69]
	Fe_3_O_4_/GO/Chitosan	Electrochemical	Bisphenol A (BPA)	6.0 × 10^−8^–1.1 × 10^−5^ M	1.7 × 10^−8^ M	Plastic powder	[70]
	S1-SA-Ab_2_-MFMGRS	Electrochemical immunoassay	Thyroxine	0.05 pg·mL^−1^–5 ng·mL^−1^	0.015 pg·mL^−1^	-	[71]
	Fe_3_O_4_/rGO-Au@Ag/Ni^2+^-Ab_2_	Electrochemical immunoassay	Carcinoembryonic antigen	0.1 pg·mL^−1^–100 ng·mL^−1^	0.0697 pg·mL^−1^	Human serum	[72]
	Fe_3_O_4_/rGO/Au	Electrochemical immunoassay	Cluster of differentiation 146 antigen (CD146)	5 pg·mL^−1^–500 ng·mL^−1^	2.5 pg·mL^−1^	Human serum	[73]
	Fe_3_O_4_/rGO-Au@Ag NPs	Electrochemical immunoassay	Human Immunoglobulin G	5 fg·L^−1^–50 ng·mL^−1^	2 fg·L^−1^	Human serum	[74]
	MGLA/poly SiNW-FET	Electronic (FET)	Apolipoprotein A II protein (APOA2 protein)	19.5 pg·mL^−1^–1.95 µg·mL^−1^	6.7 pg·mL^−1^	Human urine	[75]
	Ag@3D-Fe_3_O_4_/GO	MSPE-GC-µECD	Pesticides: Fenitrothion, Chloropyrofos, Hexaconazole	0.1–5 ng·g^−1^	0.07–0.13 ng·g^−1^	Extraction of the selected pesticides in tomato and grape samples	[76]
	Fe_3_O_4_/GO	MSPE-HPLC-UV	Methamphetamine	100–1500 ng·mL^−1^	30 ng·mL^−1^	Urine samples	[77]
In situ Hydrothermal synthesis	Fe_3_O_4_/GO-CNT	UPLC-MS	Melamine	0.0015–0.15 mg·kg^−1^	0.00045 mg·kg^−1^	Milk	[7]
	Fe_3_O_4_/GO	Colorimetric	H_2_O_2_, Glucose	1–50 × 10^−6^ M, 2–200 × 10^−6^ M	0.32 × 10^−6^ M	Diabetic urine	[78]

^1^ SERS: Surface-enhanced Raman spectroscopy.
